# PDB NextGen Archive: centralizing access to integrated annotations and enriched structural information by the Worldwide Protein Data Bank

**DOI:** 10.1093/database/baae041

**Published:** 2024-05-27

**Authors:** Preeti Choudhary, Zukang Feng, John Berrisford, Henry Chao, Yasuyo Ikegawa, Ezra Peisach, Dennis W Piehl, James Smith, Ahsan Tanweer, Mihaly Varadi, John D Westbrook, Jasmine Y Young, Ardan Patwardhan, Kyle L Morris, Jeffrey C Hoch, Genji Kurisu, Sameer Velankar, Stephen K Burley

**Affiliations:** Protein Data Bank in Europe, European Molecular Biology Laboratory, European Bioinformatics Institute Wellcome Genome Campus, Hinxton, Cambridgeshire, CB10 1SD, UK; Research Collaboratory for Structural Bioinformatics Protein Data Bank, Institute for Quantitative Biomedicine, Rutgers, The State University of New Jersey, 174 Frelinghuysen Rd., Piscataway, NJ 08854, USA; Protein Data Bank in Europe, European Molecular Biology Laboratory, European Bioinformatics Institute Wellcome Genome Campus, Hinxton, Cambridgeshire, CB10 1SD, UK; Research Collaboratory for Structural Bioinformatics Protein Data Bank, Institute for Quantitative Biomedicine, Rutgers, The State University of New Jersey, 174 Frelinghuysen Rd., Piscataway, NJ 08854, USA; Protein Data Bank Japan, Protein Research Foundation, 3-2, Yamadaoka, Minoh, Osaka 562-8686, Japan; Research Collaboratory for Structural Bioinformatics Protein Data Bank, Institute for Quantitative Biomedicine, Rutgers, The State University of New Jersey, 174 Frelinghuysen Rd., Piscataway, NJ 08854, USA; Research Collaboratory for Structural Bioinformatics Protein Data Bank, Institute for Quantitative Biomedicine, Rutgers, The State University of New Jersey, 174 Frelinghuysen Rd., Piscataway, NJ 08854, USA; Research Collaboratory for Structural Bioinformatics Protein Data Bank, Institute for Quantitative Biomedicine, Rutgers, The State University of New Jersey, 174 Frelinghuysen Rd., Piscataway, NJ 08854, USA; Protein Data Bank in Europe, European Molecular Biology Laboratory, European Bioinformatics Institute Wellcome Genome Campus, Hinxton, Cambridgeshire, CB10 1SD, UK; Protein Data Bank in Europe, European Molecular Biology Laboratory, European Bioinformatics Institute Wellcome Genome Campus, Hinxton, Cambridgeshire, CB10 1SD, UK; Research Collaboratory for Structural Bioinformatics Protein Data Bank, Institute for Quantitative Biomedicine, Rutgers, The State University of New Jersey, 174 Frelinghuysen Rd., Piscataway, NJ 08854, USA; Research Collaboratory for Structural Bioinformatics Protein Data Bank, Institute for Quantitative Biomedicine, Rutgers, The State University of New Jersey, 174 Frelinghuysen Rd., Piscataway, NJ 08854, USA; The Electron Microscopy Data Bank, European Molecular Biology Laboratory, European Bioinformatics Institute, Wellcome Genome Campus, Hinxton, Cambridgeshire, CB10 1SD, UK; The Electron Microscopy Data Bank, European Molecular Biology Laboratory, European Bioinformatics Institute, Wellcome Genome Campus, Hinxton, Cambridgeshire, CB10 1SD, UK; Biological Magnetic Resonance Data Bank, Department of Molecular Biology and Biophysics, UConn Health, 263 Farmington Avenue, Farmington, CT 06030-3305, USA; Protein Data Bank Japan, Protein Research Foundation, 3-2, Yamadaoka, Minoh, Osaka 562-8686, Japan; Protein Data Bank Japan, Institute for Protein Research, Osaka University, 3-2 Yamadaoka, Suita-shi, Osaka 565-0871, Japan; Protein Data Bank in Europe, European Molecular Biology Laboratory, European Bioinformatics Institute Wellcome Genome Campus, Hinxton, Cambridgeshire, CB10 1SD, UK; Research Collaboratory for Structural Bioinformatics Protein Data Bank, Institute for Quantitative Biomedicine, Rutgers, The State University of New Jersey, 174 Frelinghuysen Rd., Piscataway, NJ 08854, USA; Rutgers Cancer Institute of New Jersey, 195 Little Albany St., New Brunswick, NJ 08901, USA; Department of Chemistry and Chemical Biology, Rutgers, The State University of New Jersey, 123 Bevier Rd., Piscataway, NJ 08854, USA

## Abstract

The Protein Data Bank (PDB) is the global repository for public-domain experimentally determined 3D biomolecular structural information. The archival nature of the PDB presents certain challenges pertaining to updating or adding associated annotations from trusted external biodata resources. While each Worldwide PDB (wwPDB) partner has made best efforts to provide up-to-date external annotations, accessing and integrating information from disparate wwPDB data centers can be an involved process. To address this issue, the wwPDB has established the PDB Next Generation (or NextGen) Archive, developed to centralize and streamline access to enriched structural annotations from wwPDB partners and trusted external sources. At present, the NextGen Archive provides mappings between experimentally determined 3D structures of proteins and UniProt amino acid sequences, domain annotations from Pfam, SCOP2 and CATH databases and intra-molecular connectivity information. Since launch, the PDB NextGen Archive has seen substantial user engagement with over 3.5 million data file downloads, ensuring researchers have access to accurate, up-to-date and easily accessible structural annotations.

**Database URL**: http://www.wwpdb.org/ftp/pdb-nextgen-archive-site

## Introduction

The Protein Data Bank (PDB) has a remarkable history, which began in 1971 when it was established as the first open-access digital data resource in biology (1). After more than five decades of continuous operations, the PDB has grown more than 30 000-fold, increasing from 7 to more than 210 000 structures and becoming a global repository storing extensively annotated 3D structures of proteins and nucleic acids, enabling atomic-level insights into the workings of complex biological macromolecules (2). This invaluable public-domain three-dimensional (3D) structure data resource has had a profound impact on fundamental biology, biomedicine, biotechnology and bioenergy, encompassing both naturally occurring and engineered biomolecules (3, 4). Moreover, the PDB has significantly contributed to human health, facilitating discovery and development of nearly 90% of new drugs approved by the US Food and Drug Administration between 2010 and 2016 with open access to 3D biostructure information (5).

Since 2003, the PDB has been managed by the Worldwide Protein Data Bank (wwPDB) partnership, ensuring that all archival data are freely accessible without limitations on its usage (6). At that time, this international partnership brought together the RCSB Protein Data Bank (RCSB PDB) (7), Protein Data Bank in Europe (PDBe) (8) and Protein Data Bank Japan (PDBj) (9) as founding wwPDB members to oversee the PDB Core Archive, which houses atomic-level 3D structures of biological macromolecules. These experimentally determined structures are the products of macromolecular crystallography (MX), nuclear magnetic resonance (NMR) spectroscopy and electron microscopy (3DEM). Over more than two decades, the wwPDB has grown to encompass two additional full members (Biological Magnetic Resonance Data Bank or BMRB (10), responsible for archiving experimental NMR spectroscopy data; and Electron Microscopy Data Bank (EMDB) (11), responsible for archiving experimental 3DEM data) and its first associate member (PDB China or PDBc) (12). The wwPDB adheres to the principles of Fairness-Accuracy-Confidentiality-Transparency (FACT) (13) and Findability-Accessibility-Interoperability-Reusability (FAIR) (14), ensuring equitable sharing and responsible management of the 3D biostructure data. Information stored in the PDB is made available under the most permissive Creative Commons CC0 1.0 Universal License (https://creativecommons.org/licenses/by/4.0/), enabling researchers around the world to access and utilize the information. Recognizing its commitment to high standards of data management, preservation and openness, the PDB is accredited by CoreTrustSeal, an international organization that certifies data repositories (https://amt.coretrustseal.org/certificates/). More recently, the PDB was recognized by the Global Biodata Coalition (https://globalbiodata.org) as a Global Core Biodata Resource of ‘fundamental importance to the wider biological and life sciences community and the long-term preservation of biological data’.

Each PDB structure (or entry) is made up of 3D atomic coordinates, experimental data and extensive metadata, providing a wealth of information regarding sample provenance and the structure determination process. PDB metadata encompasses protein names, amino acid or nucleic acid sequences, source organism(s), small-molecule chemical information, data collection information (e.g. instrumentation) and structure-determination methodology (e.g. model-building procedures, structure quality metrics). Each PDB entry is deposited, biocurated and validated using the common global OneDep (15) software tool. During rigorous validation (16), OneDep computes a quantitative assessment of structure quality, including chemical geometry and agreement with experimental data. During biocuration (17), expert wwPDB staff members utilize OneDep (15) to incorporate sequence cross-references to trusted external biodata resources, including UniProtKB (18) or NCBI GenBank (19), which provide links to reference sequence information. Other biocuration activities support the inclusion of derived metadata, including structural characteristics (e.g. secondary and quaternary structure) and small-molecule ligand descriptors.

The PDB weekly release process collects new 3D biostructure data from each wwPDB partner, cross-checks and packages it into a pre-release FTP area and then delivers this information to wwPDB partners for distribution from regional wwPDB FTP servers. On average, more than 300 new structures are publicly released into the PDB Core Archive every Wednesday at 00:00 Universal Time Coordinated (UTC). The wwPDB FTP servers support open access to the entire contents of the PDB archive, with no login requirement or usage limitations. The downloadable information is archival in nature, faithfully reflecting the 3D biostructure data generously contributed by more than 60 000 structural biologists since 1971.

Metadata provided for the released PDB structure is primarily established by depositors or wwPDB biocurators during biocuration of a PDB entry and remains largely static thereafter. Recognizing that knowledge of biochemical, cellular and organismal context is often necessary to understand and fully utilize 3D biostructure data, the founding wwPDB partners (RCSB PDB, PDBe and PDBj) deliver additional, regularly updated functional annotations and related metadata with each PDB structure on their respective websites (rcsb.org, pdbe.org and pdbj.org, respectively). While certain annotations are specific to individual wwPDB partners, there are shared annotations, leading to redundancy of resources (Supplementary Table S1). In parallel, BMRB (https://bmrb.io/) and EMDB (https://www.ebi.ac.uk/emdb/) offer similarly valuable, regularly updated metadata for NMR and 3DEM structures, respectively. This arrangement provides valuable services to many millions of PDB data consumers around the world.

Feedback from our diverse user community, however, requested that we augment the structure data files (made available *via* FTP, HTTP and rsync) with up-to-date functional annotations and related metadata provided on the RCSB PDB, PDBe, PDBj, BMRB and EMDB web portals. Because much of the contextual information provided on these wwPDB websites is complementary, the wwPDB partnership elected to pool functional annotations and other metadata attributed to each structure and develop a ‘Next Generation’ PDB data repository, thereby providing an efficient mechanism for sharing value-added information accumulated across the wwPDB with PDB data consumers around the world whilst preserving the PDB archive as the faithful representation of the deposited information.

Herein, we describe the design and implementation of the PDB NextGen Archive, as a regularly updated (or living) one-stop-shop resource that enhances the accessibility and utilization of structural information within its biological context. The wwPDB vision for the PDB NextGen Archive is to deliver up-to-date metadata and functional annotations through an integrated and standardized methodology, thereby facilitating a comprehensive grasp of the biochemical, cellular and organismal contexts associated with protein structures. This paper presents technological advances and the collaborative efforts of the wwPDB partnership to develop the PDB NextGen Archive. It outlines data retrieval and integration mechanisms of each wwPDB partner, showcasing a dynamic, living data resource. Launch of the PDB NextGen Archive marks a significant milestone in the evolution of the PDB as a global biodata resource, giving researchers the wherewithal to explore protein structures with enriched biological context and current functional annotations. It underscores the commitment of wwPDB partners to meet the evolving needs of the biological and biomedical research and education communities and to support ground-breaking research across fundamental biology, biomedicine, biotechnology and bioenergy.

## Implementation and outcomes

Initially introduced as a successor to the legacy PDB file format, the PDB exchange/macromolecular Crystallographic Information Framework (PDBx/mmCIF) (20–22) now serves as the master format for the PDB archive, addressing the growing complexity and diversity of structural biology data (23). The PDBx/mmCIF data standard/data dictionary overcomes the limitations of the legacy PDB file format and readily accommodates PDB data encompassing very large structures of macromolecular machines and viruses, complex chemistry and new experimental methods. The benefits of the PDBx/mmCIF format are manifold. It is both human- and machine-readable. The metadata framework of the dictionary specifies data content and encompasses data typing, validation rules and organizational structures. This comprehensive approach ensures that data consistency and integrity can be maintained via automated checks. The PDBx/mmCIF data standard/data dictionary is fully extensible, permitting incorporation of new data items and categories as evidenced by extensions recently introduced to support archiving of data from X-ray Free Electron Laser/Serial Crystallography (XFEL/SX) (2). Moreover, the core PDBx/mmCIF standard promotes data sharing and interchangeability as evidenced by launch of the specialized IHMCIF (24) and ModelCIF (25) dictionaries built atop the PDBx/mmCIF data standard. By facilitating inclusion of new information and accommodating scientific advances, the PDBx/mmCIF dictionary provides enduring value to the scientific community.

Maintenance of the PDBx/mmCIF data standard/data dictionary is overseen by the wwPDB partnership, which is continuously updating it with new data elements to accommodate new science and technology. The wwPDB collaborates with the wwPDB PDBx/mmCIF Working Group, leveraging domain experts to help refine and extend the data model. This collaborative effort ensures that the PDBx/mmCIF dictionary remains relevant and readily adaptable to researchers’ evolving needs. Ongoing improvements are shared with the scientific community via the GitHub platform (https://github.com/pdbxmmcifwg), promoting transparency, collaboration and community involvement. To streamline access and utilization of PDBx/mmCIF, a dedicated data portal site (https://mmcif.wwpdb.org/) has been established. This comprehensive resource provides access to data standards, metadata specifications, tutorials and links to essential software tools. By offering these resources, the wwPDB ensures that researchers can effectively navigate and harness the myriad capabilities of PDBx/mmCIF.

Based on the PDBx/mmCIF format, the Protein Data Bank Markup Language (PDBML) supports representation of PDB data in XML format (26). This schema is the product of a direct translation of the PDBx/mmCIF Dictionary and is available for download from https://pdbml.wwpdb.org/pdbml-downloads.html. The NextGen Archive supports both PDBx/mmCIF and PDBML formats, providing researchers with two options for accessing and utilizing PDB data.

**Figure 1.**
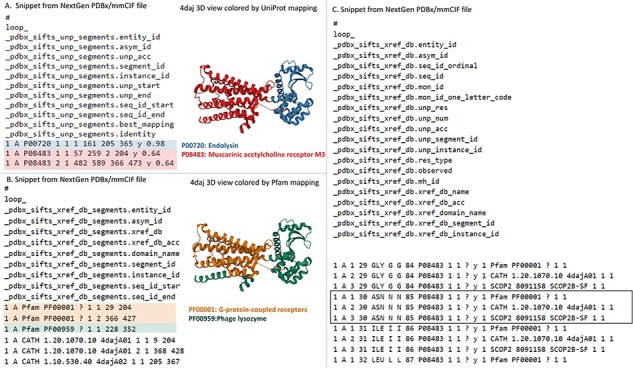
Accessing SIFTS annotations in the NextGen Archive: this figure displays a snippet from the NextGen Archive PDBx/mmCIF File for PDB ID pdb_00004daj, together with a 3D representation of the molecular structure. (A) Depicts the ‘_pdbx_sifts_unp_segments’ category, presenting two segments of PDB chain A, each mapped to UniProtKB accessions: P00720 and P08483. This suggests that PDB ID pdb_00004daj corresponds to a chimeric protein. (B) Illustrates the ‘_pdbx_sifts_xref_db_segments’ category, demonstrating residue range-based cross-references to additional databases like Pfam, SCOP2 and CATH. In this case, PDB chain A is associated with two Pfam domains, corresponding to a G-protein-coupled receptor (Pfam accession: PF00001) and Phage lysozyme (Pfam accession: PF00959). (C) Displays the ‘_pdbx_sifts_xref_db’ category, providing a comprehensive view of all mappings for each residue to external databases. Notably, the mappings from UniProt and other cross-reference databases (Pfam/SCOP2/CATH) are highlighted in a box for residue Asn30 in chain A.

**Figure 2.**
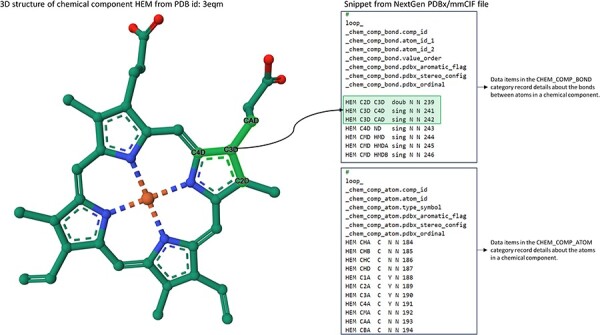
Accessing Intra-molecular Connectivity Information in NextGen Archive: this figure displays a snippet from the NextGen Archive PDBx/mmCIF File and 3D representation of Hemoglobin, identified as the chemical component CCD HEM within PDB ID 3eqm. The ‘_chem_comp_bond’ and ‘_chem_comp_atom’ categories can be used for accessing detailed information about the bonds between atoms within a chemical component and the attributes of individual atoms in that component. Notably, the image highlights a specific instance where atom C3D forms a single bond with atoms C4D and CAD, and a double bond with atom C2D.

Development of the PDB NextGen Archive prototype began with integrating the Structure Integration with Function, Taxonomy and Sequence (SIFTS) data (27, 28). This crucial step was driven by previous efforts by the PDBe team resulting in expanding the PDBx/mmCIF data dictionary to accommodate SIFTS annotations (29). This initial extension was designed to seamlessly integrate SIFTS data, which includes annotations from UniProtKB (18), Pfam (30), SCOP2 (31) and CATH (32). Value-added, residue-level annotations were directly incorporated into the PDBx/mmCIF files from the PDB Core Archive and were made available exclusively at PDB NextGen Archive. Newly introduced SIFTS-specific data categories, _pdbx_sifts_xref_unp_segments and _pdbx_sifts_xref_db_segments, offer information on PDB segments mapped to UniProt and other external databases (Pfam, SCOP2 and CATH), while _pdbx_sifts_xref_db provides a complete view of all residue mappings to these trusted external resources. For example, PDB entry 4daj (with extended PDB ID, pdb_00004daj) contains a chimeric protein that is composed of two muscarinic acetylcholine receptor M3 segments from *Rattus norvegicus* and a lysozyme segment from *Enterobacteria phage T4*. Each protein segment has mapping annotations from UniProt, Pfam and CATH resources provided in these data categories as shown in Figure 1. The _atom_site category was modified to incorporate best-mapped UniProt residue numbering. Many additional enhancements were driven by valuable insights from the PDB data consumer community. Responding to user needs, intra-molecular connectivity for each residue present in an entry was introduced. This enhancement proved instrumental in encouraging users to transition from the outdated legacy PDB format to the more powerful PDBx/mmCIF format (https://mmcif.wwpdb.org/dictionaries/mmcif_pdbx_v50.dic/Groups/reference_sequence_group). Researchers can now access the intra-molecular connectivity information by referring to the _chem_comp_bond and _chem_comp_atom categories within PDBx/mmCIF-formatted files of the PDB NextGen archive (Figure 2). (N.B.: They have also been introduced into the wwPDB FTP archive.) These categories provide comprehensive details on atom connectivity and chemical bonding to support the analysis and visualization of the molecules.

New cyber infrastructure was established to collect and combine value-added metadata, utilizing specialized services and the collective expertise of wwPDB partners. To ensure interoperability and data consistency, data enrichment processes at each wwPDB data center generate data that are fully aligned with PDBx/mmCIF semantics. These enrichment processes automatically update evolving annotations wherever this is possible. Doing so not only keeps the data up to date but also minimizes the need for manual intervention, streamlining information dissemination.

**Figure 3.**
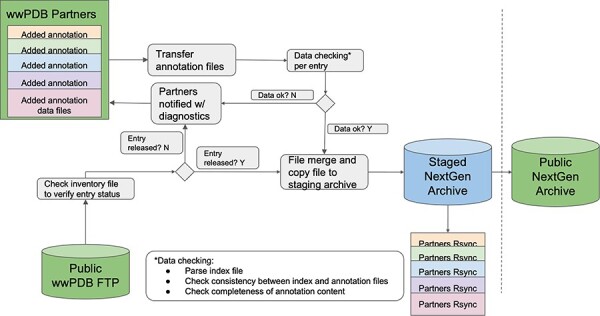
Systematic workflow of NextGen Archive: this figure outlines the structured process for maintaining and updating the NextGen Archive. It showcases key steps, including annotations collection from wwPDB partners, data quality checks, corrective actions, file aggregation and synchronized data in the staging area.

Central to this advance was the development of a common exchange area, a collaborative data hub wherein each wwPDB partner contributes annotations and metadata. At present, the wwPDB uses the Remote Synchronization (rsync) protocol to exchange data among wwPDB partners. Each wwPDB data center has a private common data exchange area, wherein data can be exchanged via rsync. Within the common exchange area, an exhaustive inventory of entries that have undergone updates for each annotation type is maintained. The inventory serves as the foundation for aggregating updated files from each wwPDB partner. As the wwPDB-designated Archive Keeper for the PDB Core Archive, RCSB PDB plays a pivotal role in maintaining all PDB archives (Main, Versioned and NextGen). A workflow was developed to collate and update the data (Figure 3). Rigorous quality checks are conducted to ensure the validity and accuracy of the merged data files: completeness of SIFTS data, PDBx/mmCIF dictionary compliance within SIFTS data file and data consistency between SIFTS data and its corresponding PDB data. In case of discrepancies or issues identified during format checking, wwPDB partners are promptly informed, and corrective actions are taken. Upon successful merging of these annotations, combined cohesive and enriched PDB data are presented in both PDBx/mmCIF and PDBML formats. Once updated/validated data are ready for distribution, they are synchronized onto a publicly accessible wwPDB NextGen archive: https://files-nextgen.wwpdb.org. This process is fully automated and is currently executed monthly. To enhance accessibility and reduce latency, NextGen archival data are mirrored by RCSB PDB, PDBe and PDBj.

**Figure 4.**
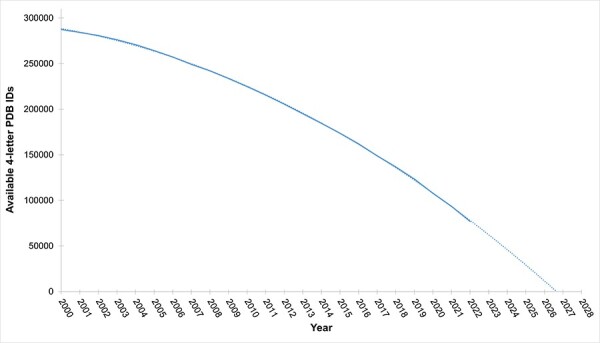
Availability of four-letter PDB codes versus time: this figure depicts the annual count of available four-letter PDB codes. Current projections anticipate exhaustion of four-letter PDB codes by the end of 2027.

With continuing growth in the size of the PDB archive, issuance of new, longer PDB IDs will become necessary as more and more structures are added (Figure 4). In anticipation of this important milestone in the history of the PDB, the wwPDB partners elected to adopt a revised PDB accession code with a prefix ‘pdb_’ and a length of eight alphanumeric characters (e.g. PDB ID 8aly will become pdb_00008aly). This new PDB ID format will offer the added benefits of enabling text mining detection of PDB structures in the scientific literature and allowing more informative and transparent delivery of revised data files. The new extended PDB ID is already being stored in most mmCIF format files under the _database_2.pdbx_database_accession data item in addition to the legacy four-character PDB ID, which is stored under _database_2.database_code. Once four-character PDB IDs are fully exhausted, the new extended PDB IDs will be represented in both _database_2.database_code and _database_2.pdbx_database_accession data items. To facilitate a more orderly transition from the legacy PDB format to the PDBx/mmCIF archival standard, NextGen file naming and data now utilize extended PDB IDs. As for the PDB Versioned archive, all data files pertaining to a particular PDB structure in the NextGen archive are stored in a single directory following a two-character hash from the penultimate two characters of the PDB code, ‘third from last character’ and ‘second from last character’. This hash code will be preserved once PDB IDs are extended to eight characters with the pdb_ prefix. Some examples are provided below:

PDB entry 8aly in the NextGen has PDB identifier pdb_00008aly and is accessible at https://files-nextgen.wwpdb.org/pdb_nextgen/data/entries/divided/al/pdb_00008aly/.

Both PDBx/mmCIF and PDBML data files are present at this location (e.g. for pdb_00008aly: pdb_00008aly_xyz-enrich.cif.gz and pdb_00008aly_xyz-no-atom-enrich.xml.gz are available respectively).

Usage of the PDB NextGen Archive is being tracked continuously to assess the breadth of impact. Between February and September 2023, more than 3.5 million enriched NextGen PDB Archive data files in PDBx/mmCIF format have been downloaded by PDB data consumers around the world. PDB NextGen Archive complements the structural data deposited in the PDB Core Archive by consolidating functional annotations, making them easily accessible to the broader scientific community. This approach ensures that data, which may be fragmented across various wwPDB partners’ websites, become more FAIR. SIFTS resource, widely employed in both research and various data resources such as UniProt, Pfam, SCOP, Kincore and COSMIC, serves to establish a common reference frame for diverse PDB structures of a specific protein based on its sequence. The integration of SIFTS residue-level mapping into PDBx/mmCIF files eliminates the need for manual renumbering of coordinate files based on protein sequence, streamlining comparative analyses across multiple PDB structures and enhancing the efficiency of functional studies. Leveraging SIFTS domain annotations from SCOP2, CATH and Pfam resources mapped to a protein sequence in PDBx/mmCIF files, researchers can easily identify the location, multiple copies and boundaries of different domains within a protein, which can help in understanding the overall structure and function of the protein. The inclusion of UniProtKB numbering facilitates effortless structural comparisons between experimental and predicted protein models. These PDBx/mmCIF files are directly compatible with various data visualization tools, simplifying the display of annotations on 3D structure views. The 3D viewer Mol* uses SIFTS UniProtKB mapping, enabling users to swiftly superimpose protein structures in their web browser. For example, the 3DMol* at RCSB PDB and PDBe websites can display structure superposition of a PDB entry with its corresponding AlphaFold predicted structure using UniProt sequence mapping directly from the atom_site in the PDBx/mmCIF file. Similarly, visualization tools like Jmol, COOT, Chimera and Eyes Open utilize intra-molecular connectivity information including atom pairs, bond order, aromatic flag and stereochemistry from these PDBx/mmCIF files in the NextGen Archive. We will continue to monitor these download trends to assess user engagement and assess impact of this archive.

## Conclusion

The incorporation of SIFTS annotations and intra-connectivity information demonstrates the potential of the NextGen Archive prototype. This will allow wwPDB partners to get feedback from the community to evaluate the utility of the new archive. The same infrastructure process for data exchange, checking and integration developed for the NextGen prototype will then be used to integrate additional annotations from other wwPDB partners. Development of the PDB NextGen Archive cyber infrastructure marks a significant milestone in PDB data integration. Through the use of a common exchange area, data enrichment, automated updates, aggregation mechanisms, rigorous quality checks and global open access, the wwPDB partnership ensures that researchers have access to accurate, up-to-date and easily accessible structural biology information, facilitating advances and discoveries across diverse scientific disciplines.

With PDBx/mmCIF-based infrastructure supporting the NextGen Archive, the extensibility of the data model and update of existing files can be achieved easily and independently from the PDB main archive. The plan for the immediate future is to further enrich annotation by providing ligand-binding information for small molecules. Understanding the function and the mechanism of action of a macromolecule in molecular detail requires study of different states or steps of the macromolecule under various conditions. Grouping-related multiple structures together with additional metadata describing the relationship and common metadata may shed light on the biology of the macromolecule(s) studied as part of an ‘investigation’ of the macromolecular machine through multiple structures representing multiple states of a macromolecular machine or multiple steps along a chemical reaction pathway. wwPDB plans to extend the PDBx/mmCIF data dictionary to enable additional descriptive metadata and management of grouped 3D structures and deliver them at PDB NextGen Archive as investigational data packages to researchers, educators and students.

The NextGen Archive update schedule is currently monthly.

The wwPDB partnership strongly encourages scientific journals to adopt the new PDB ID format (‘pdb_’ prefix followed by eight alphanumeric characters) as soon as possible. Existing entries with four-character PDB IDs are given new PDB IDs by adding prefixing ‘pdb_0000’ to the four-character IDs when entries are updated (e.g. the new extended identifier for PDB ID ‘1abc’ is ‘pdb_00001abc’). Update of existing entries with extended PDB IDs in the _database category will be completed by the end of 2024.

## Usage notes

The PDB NextGen Archive files are accessible via wwPDB HTTPS and rsync protocols and its mirrors in the USA, UK and Japan via the following links, respectively:

- wwPDB: https://files-nextgen.wwpdb.org, rsync://rsync-nextgen.wwpdb.org
- RCSB PDB (USA): https://files-nextgen.rcsb.org, rsync://rsync-nextgen.rcsb.org
- PDBe (UK): http://ftp.ebi.ac.uk/pub/databases/pdb_nextgen
- PDBj (Japan): https://files-nextgen.pdbj.org, rsync://rsync-nextgen.pdbj.org

At these locations, both PDBx/mmCIF and PDBML format files are provided, with the suffix ‘_xyz-no-atom-enrich.xml’ for PDBML and ‘_xyz-enrich.cif’ for PDBx/mmCIF. More download mechanisms and additional details can be found on the official download page: https://www.wwpdb.org/ftp/pdb-nextgen-archive-site.

The PDB NextGen Repository is currently updated on the first Wednesday of each month at 00:00 UTC.

## Supplementary Material

baae041_Supp

## References

[1] (1971) Crystallography: Protein Data Bank. *Nat. New Biol*., 233, 223.

[2] wwPDB consortium . (2019) Protein Data Bank: the single global archive for 3D macromolecular structure data. *Nucleic Acids Res*., 47, D520–D528. 30357364 10.1093/nar/gky949 PMC6324056

[3] Burley S.K. , Berman H.M., Bhikadiya C. et al. (2019) RCSB Protein Data Bank: biological macromolecular structures enabling research and education in fundamental biology, biomedicine, biotechnology and energy. *Nucleic Acids Res*., 47, D464–D474. 30357411 10.1093/nar/gky1004 PMC6324064

[4] Goodsell D.S. , Zardecki C., Di Costanzo L. et al. (2020) RCSB Protein Data Bank: enabling biomedical research and drug discovery. *Protein Sci. Publ. Protein Soc*., 29, 52–65. 10.1002/pro.3730 PMC6933845 31531901

[5] Westbrook J.D. and Burley S.K. (2019) How structural biologists and the Protein Data Bank contributed to recent FDA new drug approvals. *Struct. Lond. Engl. 1993*, 27, 211–217. 10.1016/j.str.2018.11.007 PMC7325526 30595456

[6] Berman H. , Henrick K. and Nakamura H. (2003) Announcing the worldwide Protein Data Bank. *Nat. Struct. Biol*., 10, 980. 10.1038/nsb1203-980 14634627

[7] Berman H.M. , Westbrook J., Feng Z. et al. (2000) The Protein Data Bank. *Nucleic Acids Res*., 28, 235–242. 10592235 10.1093/nar/28.1.235 PMC102472

[8] Velankar S. , van Ginkel G., Alhroub Y. et al. (2016) PDBe: improved accessibility of macromolecular structure data from PDB and EMDB. *Nucleic Acids Res*., 44, D385–D395. 26476444 10.1093/nar/gkv1047 PMC4702783

[9] Kinjo A.R. , Suzuki H., Yamashita R. et al. (2012) Protein Data Bank Japan (PDBj): maintaining a structural data archive and resource description framework format. *Nucleic Acids Res*., 40, D453–D460. 21976737 10.1093/nar/gkr811 PMC3245181

[10] Berman H. , Henrick K., Nakamura H. et al. (2007) The worldwide Protein Data Bank (wwPDB): ensuring a single, uniform archive of PDB data. *Nucleic Acids Res*., 35, D301–D303. 17142228 10.1093/nar/gkl971 PMC1669775

[11] Lawson C.L. , Patwardhan A., Baker M.L. et al. (2016) EMDataBank unified data resource for 3DEM. *Nucleic Acids Res*., 44, D396–D403. 26578576 10.1093/nar/gkv1126 PMC4702818

[12] Xu W. , Velankar S., Patwardhan A. et al. (2023) Announcing the launch of Protein Data Bank China as an associate member of the worldwide Protein Data Bank partnership. *Acta Crystallogr. Sect. Struct. Biol*., 79, 792–795. 10.1107/S2059798323006381 PMC10478634 37561405

[13] van der Aalst W.M.P. , Bichler M. and Heinzl A. (2017) Responsible data science. *Bus. Inf. Syst. Eng*., 59, 311–313.

[14] Wilkinson M.D. , Dumontier M., Aalbersberg I.J. et al. (2016) The FAIR guiding principles for scientific data management and stewardship. *Sci. Data*, 3, 160018–160026. 26978244 10.1038/sdata.2016.18 PMC4792175

[15] Young J.Y. , Westbrook J.D., Feng Z. et al. (2017) OneDep: unified wwPDB system for deposition, biocuration, and validation of macromolecular structures in the PDB archive. *Structure*, 25, 536–545. 28190782 10.1016/j.str.2017.01.004 PMC5360273

[16] Gore S. , Sanz García E., Hendrickx P.M.S. et al. (2017) Validation of structures in the Protein Data Bank. *Struct. Lond. Engl. 1993*, 25, 1916–1927. 10.1016/j.str.2017.10.009 PMC5718880 29174494

[17] Young J.Y. , Westbrook J.D., Feng Z. et al. (2018) Worldwide Protein Data Bank biocuration supporting open access to high-quality 3D structural biology data. *Database*, 2018, bay002. 10.1093/database/bay002 PMC5804564 29688351

[18] The UniProt Consortium . (2023) UniProt: the Universal Protein Knowledgebase in 2023. *Nucleic Acids Res*., 51, D523–D531. 36408920 10.1093/nar/gkac1052 PMC9825514

[19] Sayers E.W. , Cavanaugh M., Clark K. et al. (2023) GenBank 2023 update. *Nucleic Acids Res*., 51, D141–D144. 36350640 10.1093/nar/gkac1012 PMC9825519

[20] Fitzgerald P.M.D. , Berman H., Bourne P. et al. (1996) The mmCIF dictionary: community review and final approval. *Acta Crystallogr. Sect. A*, 52, C575.

[21] Westbrook J. , Henrick K., Ulrich E. et al. (2005) Appendix 3.6. 2. The Protein Data Bank exchange data dictionary. *Int. Tables Crystallogr. Defin. Exch. Crystallogr. Data Springer Dordr. Neth*. G, 195–198.

[22] Westbrook J.D. and Fitzgerald P.M.D. (2003) The PDB format, mmCIF, and other data formats. *Methods Biochem. Anal*., 44, 161–179. 12647386

[23] Westbrook J.D. , Young J.Y., Shao C. et al. (2022) PDBx/mmCIF ecosystem: foundational semantic tools for structural biology. *J. Mol. Biol*., 434, 167599–167611. 35460671 10.1016/j.jmb.2022.167599 PMC10292674

[24] Vallat B. , Webb B., Fayazi M. et al. (2021) New system for archiving integrative structures. *Acta Crystallogr. Sect. D*, 77, 1486–1496. 10.1107/S2059798321010871 PMC8647179 34866606

[25] Vallat B. , Tauriello G., Bienert S. et al. (2023) ModelCIF: an extension of PDBx/mmCIF data representation for computed structure models. *Comput. Res. Mol. Biol*., 435, 168021–168029. 10.1016/j.jmb.2023.168021 PMC10293049 36828268

[26] Westbrook J. , Ito N., Nakamura H. et al. (2005) PDBML: the representation of archival macromolecular structure data in XML. *Bioinformatics*, 21, 988–992. 15509603 10.1093/bioinformatics/bti082

[27] Velankar S. , Dana J.M., Jacobsen J. et al. (2013) SIFTS: structure integration with function, taxonomy and sequences resource. *Nucleic Acids Res*., 41, D483–D489. 23203869 10.1093/nar/gks1258 PMC3531078

[28] Dana J.M. , Gutmanas A., Tyagi N. et al. (2019) SIFTS: updated structure integration with function, taxonomy and sequences resource allows 40-fold increase in coverage of structure-based annotations for proteins. *Nucleic Acids Res*., 47, D482–D489. 30445541 10.1093/nar/gky1114 PMC6324003

[29] Choudhary P. , Anyango S., Berrisford J. et al. (2023) Unified access to up-to-date residue-level annotations from UniProtKB and other biological databases for PDB data. *Sci. Data*, 10, 204–216. 37045837 10.1038/s41597-023-02101-6 PMC10097656

[30] Mistry J. , Chuguransky S., Williams L. et al. (2021) Pfam: the protein families database in 2021. *Nucleic Acids Res*., 49, D412–D419. 33125078 10.1093/nar/gkaa913 PMC7779014

[31] Andreeva A. , Kulesha E., Gough J. et al. (2020) The SCOP database in 2020: expanded classification of representative family and superfamily domains of known protein structures. *Nucleic Acids Res*., 48, D376–D382. 31724711 10.1093/nar/gkz1064 PMC7139981

[32] Sillitoe I. , Bordin N., Dawson N. et al. (2021) CATH: increased structural coverage of functional space. *Nucleic Acids Res*., 49, D266–D273. 33237325 10.1093/nar/gkaa1079 PMC7778904

