# Supplementary material for: PDB NextGen Archive: centralizing access to integrated annotations and enriched structural information by the Worldwide Protein Data Bank
Source: Database (Oxford). 2024 May 27;2024:baae041. doi: 10.1093/database/baae041 (PMC11130521; doi:10.1093/database/baae041)
Supplement: baae041_Supp [file baae041_supp.zip › suppl_data/Supplementary_table_DATABASE-2023-0141.pdf]

S.No.	Resource	Details of resource	PDBe	RCSB PDB	PDBj
1	14-3-3-pred	14-3-3-binding sites	YES	NO	NO
2	3DComplex	Biologically relevant interfaces	YES	NO	NO
3	3DLigandSite	Ligand binding sites prediction	YES	NO	NO
4	AKID	PTM site prediction	YES	NO	NO
5	AlphaFold DB	Computed Structure Models by AlphaFold2	YES	YES	NO
6	Arpeggio	Calculations of interatomic interactions in protein structures	YES	NO	NO
7	ATC	Anatomical Therapeutic Chemical (ATC) Classification System from World Health Organization	NO	YES	NO
8	Binding MOAD	Binding affinities	NO	YES	YES
9	BindingDB	Binding affinities	NO	YES	NO
10	BMRB	BMRB-to-PDB mappings	YES	YES	YES
11	BSM-Arc	Archive of computationally derived data related to structural biophysics	NO	NO	YES
12	CamKinet	Curated CAM-kinase sites	YES	NO	NO
13	canSAR	Druggable sites prediction	YES	NO	NO
14	CATH	Protein structure classification- Class, Architecture, Topology/fold, and Homologous superfamily	YES	YES	YES
15	CATH-FunSites	Conserved sites prediction	YES	NO	NO
16	ChannelsDB	Transport channel prediction	YES	NO	NO
17	ChEBI	Chemical entities of biological interest	YES	YES	NO
18	ChEMBL	Manually curated database of bioactive molecules with drug-like properties	YES	YES	NO
19	Complex Portal	manually curated macromolecular complexes	YES	NO	NO
20	COSPI-Depth	Residue depth	YES	NO	NO
21	Covalentizer	Candidates for covalent analogues of small molecules	YES	NO	NO
22	CSD	Cambridge Structural Database: Validated and curated small-molecule organic and metal-organic crystal structures from the Cambridge Crystallographic Data Centre	YES	YES	YES
23	DDBJ/EMBL	EMBL-Bank nucleotide sequence database (hosted at DDBJ)	NO	NO	YES
24	DrugBank	Drug and drug target data	YES	YES	NO
25	DynaMine	protein backbone dynamics	YES	NO	NO
26	ECOD	Evolutionary Classification of Protein Domains	NO	YES	NO
27	EFoldMine	Residues that start to fold independently in proteins	YES	NO	NO
28	eF-site	Database of 3D electrostatic surfaces	NO	NO	YES
29	EMDB	3DEM density maps and associated metadata	YES	YES	YES

S.No.	Resource	Details of resource	PDBe	RCSB PDB	PDBj
30	EMPIAR	Electron Microscopy Public Image Archive	NO	NO	YES
31	EMVS	Different quality and validation scores about cryo-Electron Microscopy maps and their corresponding atomic models.	YES	NO	NO
32	ExPASy	Expasy enzyme database	NO	NO	YES
33	ExplorEnz	IUBMB Enzyme nomenclature and classification	NO	YES	NO
34	EzCatDB	Database of Enzyme Catalytic Mechanisms	NO	NO	YES
35	FireProtDB	Stability of single-point mutants	YES	NO	NO
36	FoldX	interactions contributing to the stability of proteins and protein complexes	YES	NO	NO
37	Frustratometer	Delta Frustration between the native and the mutated variant for the PDBs where the mutations are located.	YES	NO	NO
38	GenBank	Annotated collection of all publicly available DNA sequences	NO	NO	YES
39	GenBank/GenPept	Annotated coding regions in GenBank	NO	NO	YES
40	Gencode	Human and Mouse Gene annotations	NO	YES	NO
41	Gene Ontology	Organization of biological data related to molecular functions, cellular components, and biological processes	YES	YES	YES
42	Genotype-Tissue Expression - GTEx (NIH Common Fund Data Resource)	Tissue-specific gene expression data	NO	YES	NO
43	GlyCosmos	A web portal aiming to integrate the glycosciences with the life sciences about genes, proteins, lipids, pathways and diseases.	NO	YES	NO
44	GlyGen	A data integration and dissemination resource for carbohydrate and glycoconjugate related data which provides computational and informatics resources and tools for glycosciences research.	NO	YES	NO
45	GlyTouCan	An international glycan structure repository with unique accession code for each unique glycan.	NO	YES	YES
46	Human Gene Nomenclature Committee	Human gene name nomenclature and genomic information	NO	YES	NO
47	Ideal	experimentally verified IDPs	NO	NO	YES
48	IMGT	The international ImMunoGeneTics information system	NO	YES	NO
49	Immune Epitope Database	Antibody and T cell epitopes	NO	YES	NO
50	International Mouse Phenotyping Consortium - IMPC (NIH Common Fund Data Resource)	Mouse gene phenotype data	NO	YES	NO

S.No.	Resource	Details of resource	PDBe	RCSB PDB	PDBj
51	InterPro	Classification of protein families	YES	YES	NO
52	IUBMB	IUBMB enzyme database	NO	NO	YES
53	KEGG	Database resource for understanding high-level functions and utilities of the biological system	NO	NO	YES
54	KinCore	Conformational annotations for protein kinase structures	YES	NO	NO
55	KnotProt	Knots or slipknots on protein chains	YES	NO	NO
56	ligandbox	Database of small chemical compound structures that are ready-to-dock	NO	NO	YES
57	M-CSA	Curated catalytic sites	YES	NO	YES
58	MemProtMD	Annotation of transmembrane protein structures	NO	YES	NO
59	MetalPDB	Curated metal binding sites	YES	NO	NO
60	Missense3D	predicted structural changes introduced by an amino acid substitution	YES	NO	NO
61	MMDB	Database of similar structures	NO	NO	YES
62	MobiDB	Intrinsic disorder predictions	YES	NO	NO
63	ModelArchive	Computed Structure Models (e.g., by RoseTTAFold)	YES	YES	NO
64	mpstruc	Classification of transmembrane protein structures	NO	YES	NO
65	NCBI Gene	Gene info, reference sequences, et al.	YES	YES	YES
66	NCBI Taxonomy	Organism Classification	YES	YES	YES
67	NDB	Experimentally-determined nucleic acids and complex assemblies	NO	YES	NO
68	Norine	Database of nonribosomal peptides	NO	NO	YES
69	OpenCitations	Citation count for articles	NO	NO	YES
70	OPM	Classification of transmembrane protein structures and membrane segments	NO	YES	NO
71	P2Rank	Pocket prediction	YES	NO	NO
72	PDBbind-CN	Binding affinities	NO	YES	NO
73	PDBflex	Protein structure flexibility	NO	YES	NO
74	PDB-REDO	optimised versions of existing PDB entries with electron density maps, a description of model changes, and a wealth of model validation data.	YES	NO	NO
75	PDBSum	pictorial database that provides an at-a-glance overview of the contents of each 3D structure deposited in the Protein Data Bank	YES	NO	NO
76	PDBTM	Annotation of transmembrane protein structures and membrane segments	YES	YES	NO
77	Pfam	Protein families	YES	YES	YES

S.No.	Resource	Details of resource	PDBe	RCSB PDB	PDBj
78	Pharos - Illuminating the Druggable Genome (NIH Common Fund Data Resource)	Drug targets and diseases	NO	YES	NO
79	POPSCOMP	Surface accessibility	YES	NO	NO
80	ProKinO	Curated PTM sites	YES	NO	NO
81	ProMode Elastic	Database of protein dynamics by normal mode analysis	NO	NO	YES
82	PROSITE	Database of protein domains, families and functional sites	NO	NO	YES
83	ProteinDiffraction.org	Diffraction images	NO	YES	NO
84	Proteopedia	free, collaborative 3D-encyclopedia of proteins & other biomolecules.	YES	NO	NO
85	PubChem	Chemical information	YES	YES	NO
86	PubMed	Citation information	YES	YES	YES
87	PubMedCentral	Open access literature	NO	YES	NO
88	PyDISH	Database of heme porphyrin structures	NO	NO	YES
89	RESID	Protein modifications	NO	YES	NO
90	SAAPpred	Structural effects of a mutation	YES	NO	NO
91	SAbDab	The Structural Antibody Database	NO	YES	NO
92	SASBDB	curated a repository for small angle X-ray scattering (SAXS) and neutron scattering (SANS) data and derived models.	YES	NO	NO
93	SBGrid	Structural Biology Data Grid / diffraction images	NO	YES	NO
94	SCOP	Structural Classification of Proteins	YES	YES	YES
95	Scop3P	Human phosphorylation sites	YES	NO	NO
96	SCOPe	Structural Classification of Proteins - extended	NO	YES	NO
97	SIFTS	Structure Integration with Function, Taxonomy and Sequence	YES	YES	YES
98	SKEMPI	binding free energy changes upon mutation	YES	NO	NO
99	Thera-SAbDab	Therapeutic Structural Antibody Database	NO	YES	NO
100	UniProt	Protein sequences and annotations	YES	YES	YES
101	WEBnma	Flexibility prediction	YES	NO	NO
102	XRDA	Xtal Raw Data Archive	NO	NO	YES
